# Severe Inflammatory Cystitis and Ureteritis Following Neoadjuvant TCHP (Docetaxel, Carboplatin, Trastuzumab, and Pertuzumab) Therapy in HER2-Positive Breast Cancer: A Case Report and Literature Review

**DOI:** 10.7759/cureus.110454

**Published:** 2026-06-08

**Authors:** Navin Mathiyalagan, Eric Rahul, Muhammad Adeel Sarwar, Eiman Abdelmoneim Ali, Sarah Khan

**Affiliations:** 1 Medical Oncology, Nottingham City Hospital, Nottingham, GBR

**Keywords:** carboplatin, cystitis, haematuria, her2-positive breast cancer, pertuzumab, tchp, trastuzumab, ureteritis

## Abstract

Haematuria is a rare complication in patients receiving docetaxel, carboplatin, trastuzumab, and pertuzumab (TCHP) chemotherapy for breast cancer, with only a few cases reported. Here, we describe a 65-year-old woman with HER2-positive T2N1 breast cancer who developed visible haematuria and acute kidney injury (AKI) after her first cycle of neoadjuvant TCHP therapy. Ultrasound and computed tomography (CT) imaging of the renal tract demonstrated mild bilateral hydronephrosis and moderate bilateral hydroureter associated with inflammatory fat stranding, with findings most consistent with chemotherapy-associated inflammatory ureteritis and cystitis. Initial management included continuous bladder irrigation, intravenous fluids, and empirical antibiotic therapy. However, persistent haematuria and worsening renal function despite supportive treatment prompted initiation of oral prednisolone 60 mg once daily for presumed inflammatory urothelial toxicity. The patient showed a marked clinical response within 72 hours of corticosteroid initiation, with resolution of haematuria and recovery of renal function to baseline. Following multidisciplinary team (MDT) discussion, and given poor tolerance of systemic therapy alongside significant treatment-related anxiety, a decision was made to proceed directly to surgery rather than continue neoadjuvant chemotherapy. This case highlights chemotherapy-associated urothelial toxicity as a rare but clinically significant adverse effect of TCHP therapy and the need for early recognition and multidisciplinary management. A focused review of previously reported cases is also presented.

## Introduction

Urothelial toxicity resulting from systemic anticancer therapy is an uncommon yet potentially serious complication. Clinical manifestations may include cystitis, ureteritis, haematuria, obstructive uropathy, or upper urinary tract inflammation. Among chemotherapeutic agents, haemorrhagic cystitis is most frequently associated with oxazaphosphorine alkylating agents, such as cyclophosphamide and ifosfamide [1,2]. In these instances, the toxic metabolite acrolein induces oxidative stress and the release of inflammatory mediators, leading to urothelial oedema, mucosal injury, and haemorrhage [2].

Docetaxel, carboplatin, trastuzumab, and pertuzumab (TCHP) is a commonly used neoadjuvant regimen for HER2-positive breast cancer and is associated with high pathological complete response rates and improved long-term outcomes [3-5]. Docetaxel is a taxane that stabilises microtubules and inhibits cell division; carboplatin is a platinum-based agent that induces DNA cross-linking and cellular apoptosis; trastuzumab and pertuzumab are HER2-targeted monoclonal antibodies that inhibit HER2 signalling through complementary mechanisms and enhance antitumour activity [3-5]. The toxicity profiles of these agents are well established and typically include myelosuppression, gastrointestinal toxicity, peripheral neuropathy, infusion-related reactions, and cardiotoxicity [4,5]. By contrast, significant urothelial toxicities, including inflammatory cystitis, ureteritis, and severe haematuria, are rarely reported with this regimen [6-9].

To date, only a limited number of cases of haematuria or urothelial toxicity associated with TCHP therapy or its constituent agents have been reported [6-9]. In previously reported TCHP-associated cases, docetaxel has been proposed as the most likely causative agent, although definitive attribution remains challenging in the setting of combination therapy [6]. The rarity of these presentations makes diagnosis and management particularly challenging, especially because more common causes of haematuria and urinary tract inflammation, including infection, obstructive uropathy, and malignancy, must first be excluded. Distinguishing drug-induced urothelial toxicity from these alternative diagnoses is important, as management strategies and decisions regarding continuation of anticancer therapy may differ substantially.

This report presents a rare case of severe inflammatory cystitis and bilateral ureteritis following neoadjuvant TCHP chemotherapy in a patient with HER2-positive breast cancer, resulting in gross haematuria, acute kidney injury, and bilateral hydroureteronephrosis. We also review previously reported cases and discuss possible mechanisms and management considerations.

## Case presentation

**Figure 1:**
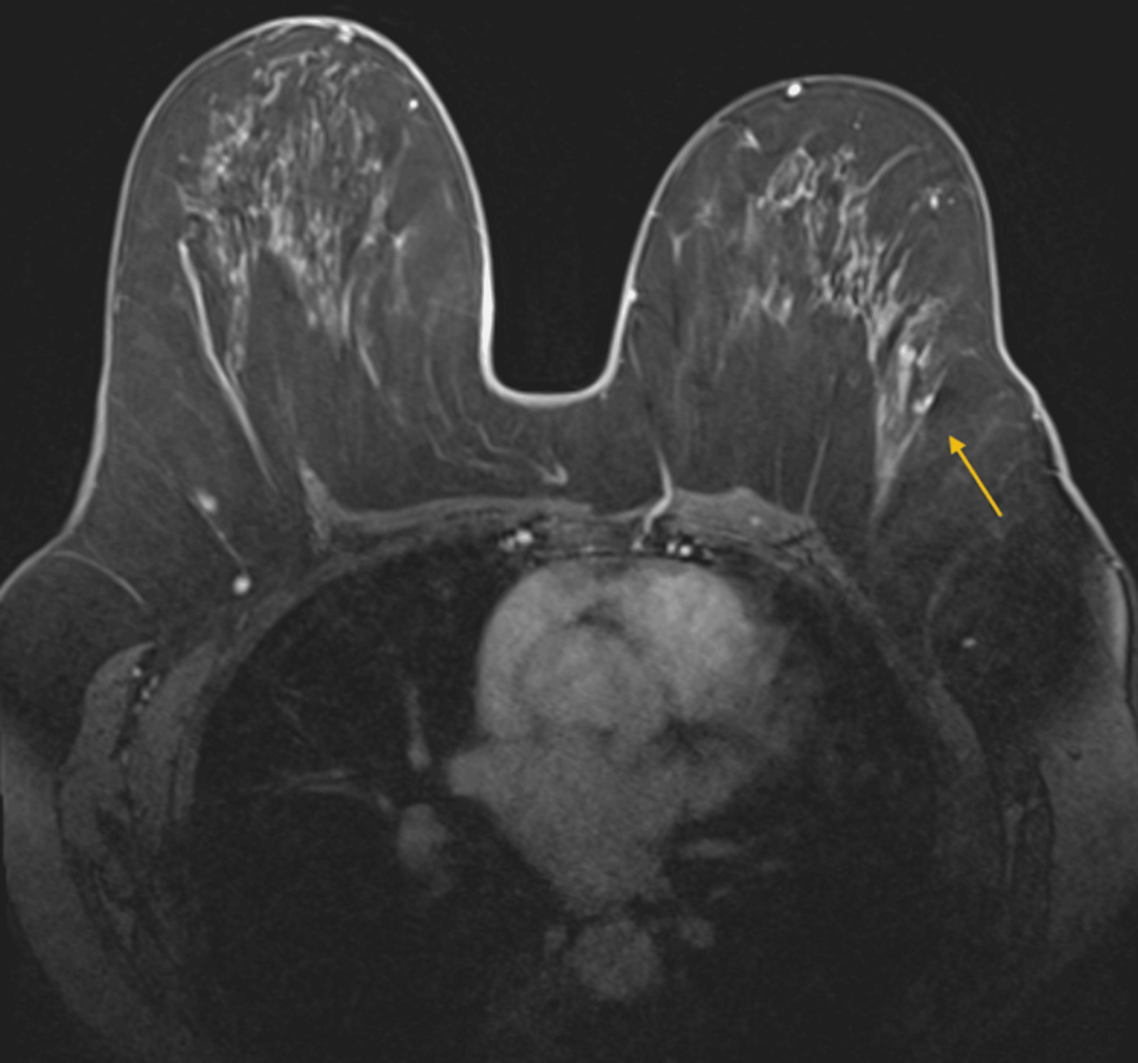
MRI of the breasts showing left breast cancer in the upper outer quadrant (yellow arrow)

**Figure 2:**
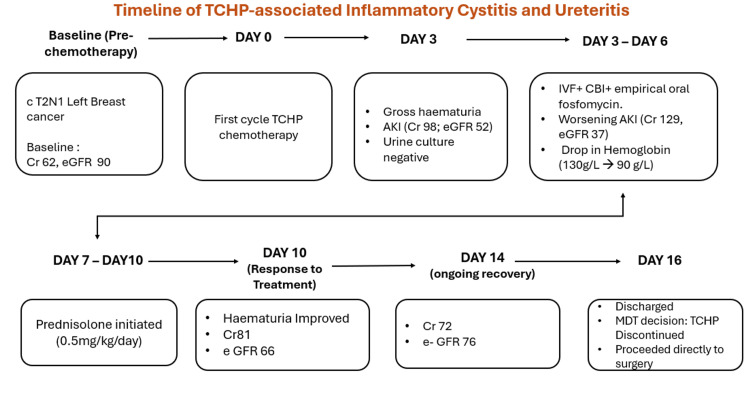
Timeline of clinical presentation, investigations, management, and outcome of TCHP- associated inflammatory cystitis and ureteritis Abbreviations: TCHP, docetaxel, carboplatin, trastuzumab, and pertuzumab; AKI, acute kidney injury; Cr, serum creatinine; eGFR, estimated glomerular filtration rate; IVF, intravenous fluids; CBI, continuous bladder irrigation; MDT, multidisciplinary team

A 65-year-old woman with no other significant medical history presented to the emergency department with abdominal pain and visible haematuria. Symptoms developed shortly after completing her first cycle of neoadjuvant chemotherapy (docetaxel, carboplatin, trastuzumab, and pertuzumab) for HER2-positive, hormone receptor-negative T2N1 left breast cancer (Figure 1). A summary of the clinical course, investigations, treatment, and outcome is provided in Figure 2.

On arrival, she was haemodynamically stable. Blood tests demonstrated stage 1 acute kidney injury, with serum creatinine increasing from a baseline of 62 µmol/L (estimated glomerular filtration rate (eGFR) >90 mL/min/1.73 m²) to 98 µmol/L (eGFR 52 mL/min/1.73 m²). Urine microscopy, culture, and sensitivity revealed significant haematuria and pyuria without bacterial growth. She was initially managed with intravenous fluids and supportive care. There was no evidence of coagulopathy, thrombocytopenia, nephrolithiasis, or urinary tract malignancy.

The urology team recommended continuous bladder irrigation with a three-way catheter and outpatient flexible cystoscopy. Empirical oral fosfomycin was started. She was then transferred to the oncology ward for ongoing care.

**Figure 3:**
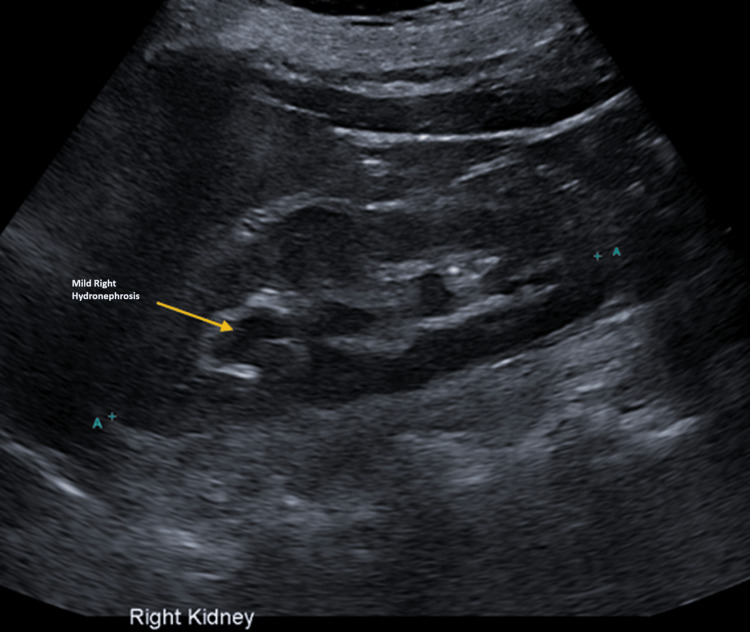
Ultrasound image of the right kidney demonstrating mild pelvicalyceal dilatation (hydronephrosis) (yellow arrow)

**Figure 4:**
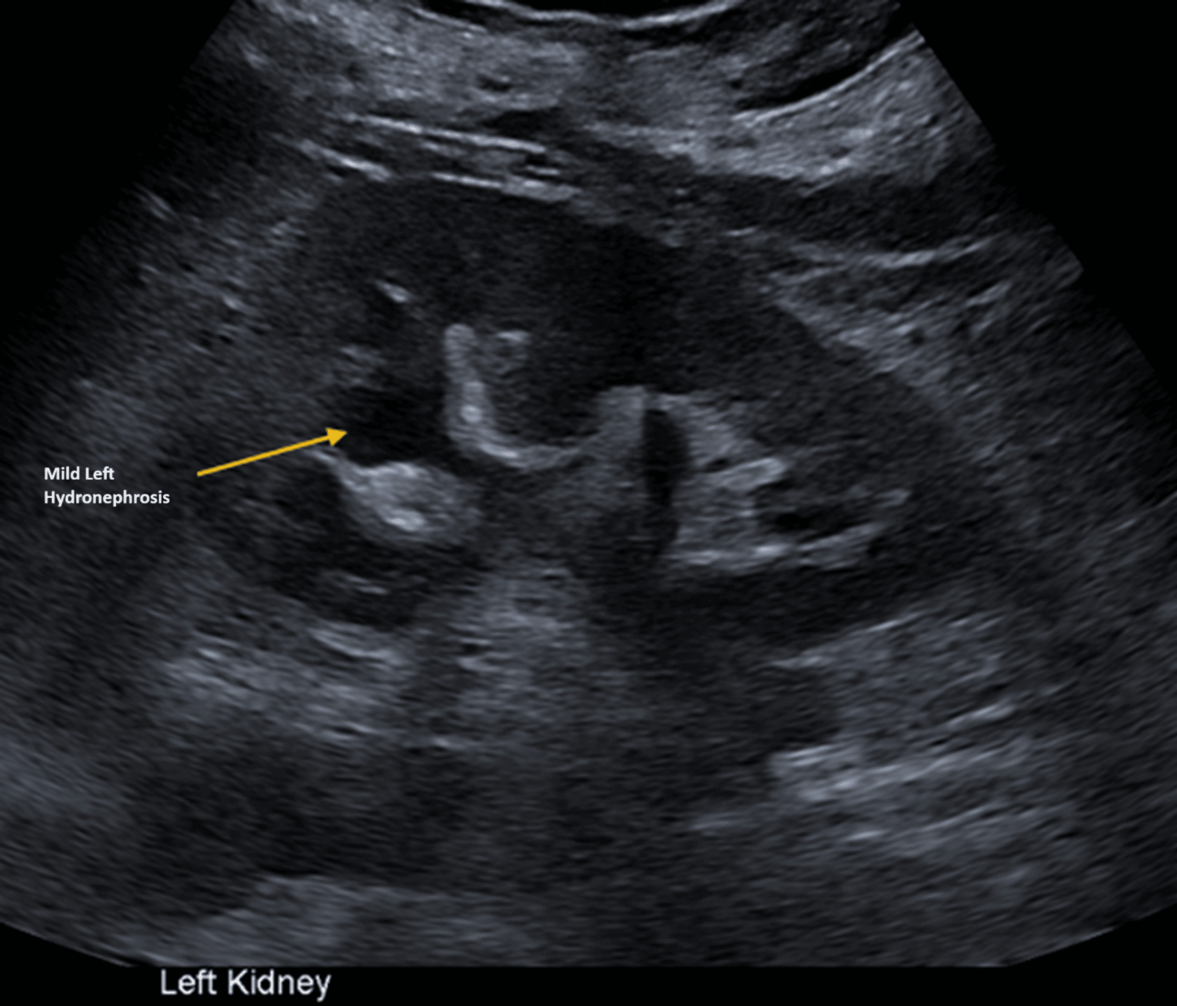
Ultrasound image of the left kidney demonstrating mild pelvicalyceal dilatation (hydronephrosis) (yellow arrow)

**Figure 5:**
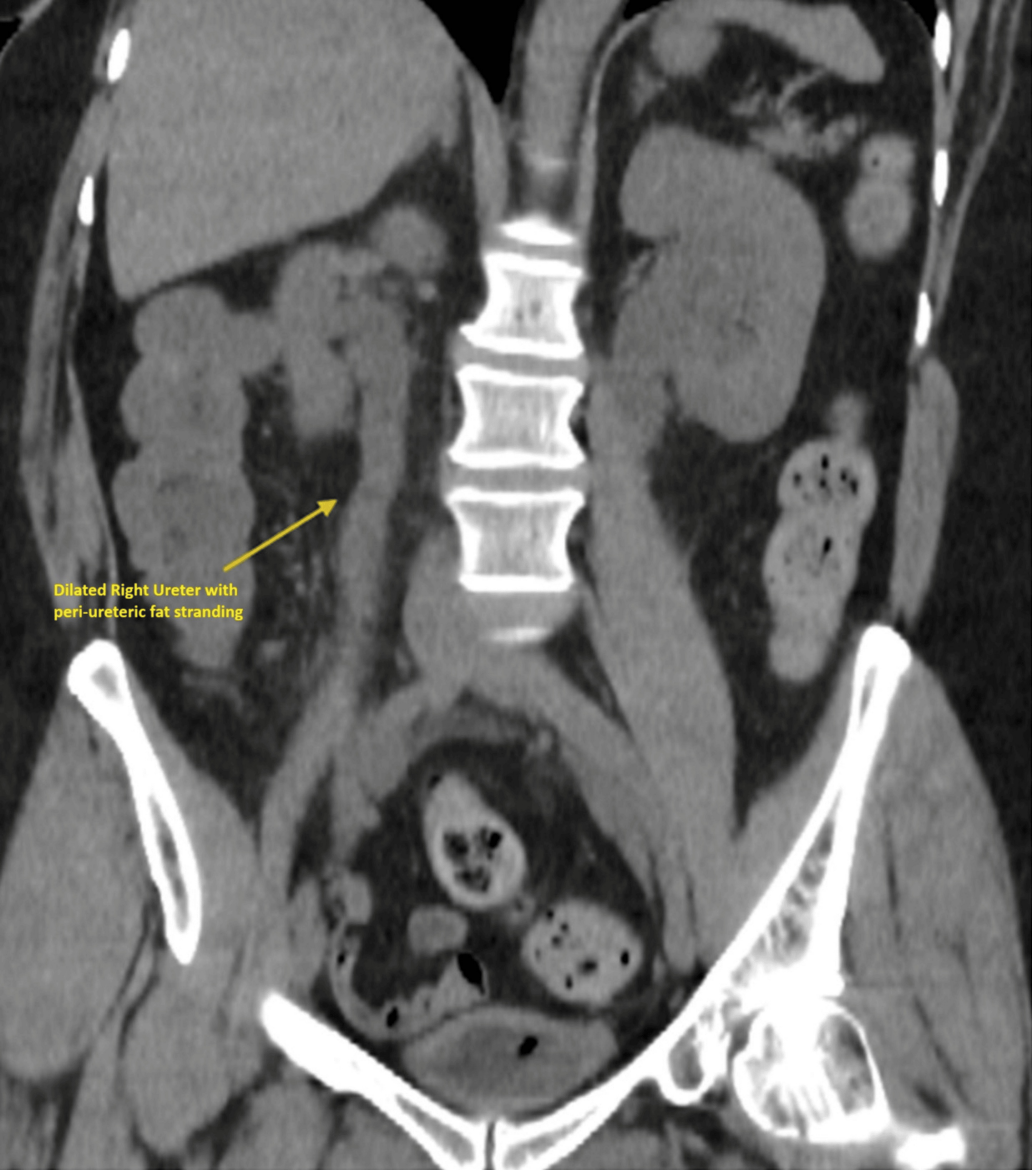
Coronal CT renal tract image showing dilatation of the right ureter with adjacent peri-ureteric inflammatory fat stranding (yellow arrow)

**Figure 6:**
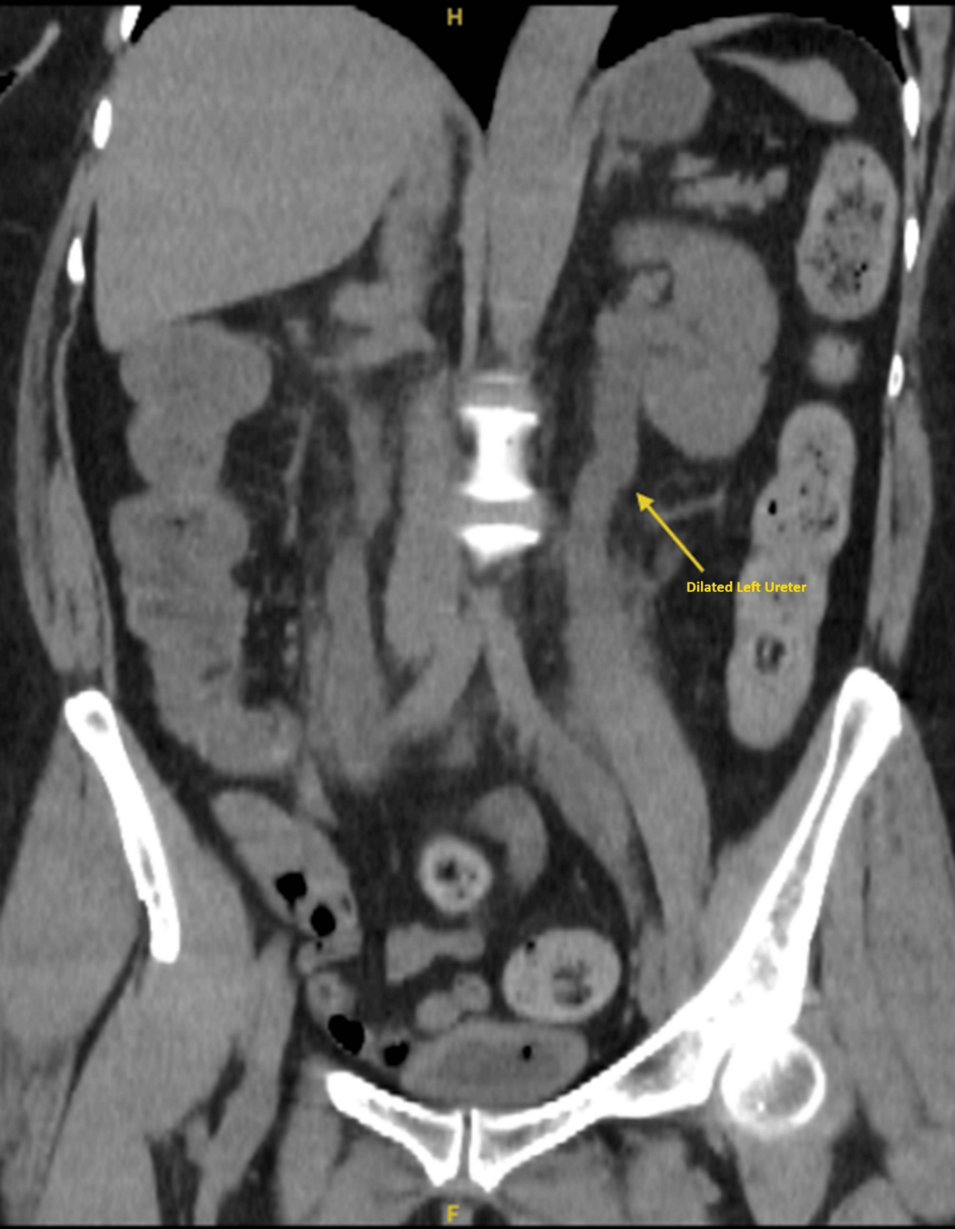
Coronal CT renal tract image demonstrating dilatation of the left ureter (yellow arrow)

Ultrasound KUB demonstrated bilateral hydronephrosis involving both kidneys (Figures 3, 4). CT renal tract imaging demonstrated moderate bilateral hydroureter with associated peri-ureteric inflammatory fat stranding, more prominent on the right side (Figures 5, 6). No obstructing renal or ureteric calculi were identified. The radiological findings were most consistent with inflammatory ureteritis.

Despite supportive intravenous fluids, serial blood tests demonstrated worsening renal function and persistent haematuria. Serum creatinine increased from 98 µmol/L to 129 µmol/L, corresponding to a decline in eGFR from 52 to 37 mL/min/1.73 m², and haemoglobin fell from 130 g/L to 90 g/L. After multidisciplinary discussion with renal and urology teams, oral prednisolone 60 mg once daily (0.5 mg/kg/day) was commenced for presumed treatment-related inflammatory cystitis and ureteritis. At the time of treatment initiation, urine cultures remained negative, and no alternative obstructive or malignant cause was identified on imaging. Given the severity of haematuria, associated ureteritis with bilateral hydroureteronephrosis, and worsening renal impairment, corticosteroid therapy was initiated empirically. In the absence of established guidelines for TCHP-associated urothelial toxicity, a moderate-dose corticosteroid regimen (0.5 mg/kg/day) was selected to suppress the presumed inflammatory process while balancing the risks associated with higher-dose corticosteroid therapy. Renal biopsy was not recommended, as the overall clinical and radiological findings were felt to be most consistent with a treatment-related inflammatory urothelial process, and the result was not expected to alter immediate management. Although alternative diagnoses such as drug-induced interstitial nephritis, IgA nephropathy, or vasculitis could not be completely excluded in the absence of tissue diagnosis, the multidisciplinary team (MDT) considered these less likely given the temporal relationship to TCHP administration, the radiological findings, and the absence of an alternative explanation.

A marked clinical improvement was observed within 72 hours of commencing corticosteroid therapy, including reduced haematuria, improved renal function, and stabilisation of haemoglobin levels. Renal function returned to baseline within seven days of starting prednisolone. Steroids were tapered by 10 mg every five days, with close monitoring for recurrence of haematuria or renal deterioration.

Continuous bladder irrigation was discontinued once the urine cleared, and the catheter was removed following a successful trial without a catheter. Subsequent outpatient flexible cystoscopy, performed after resolution of the acute episode, demonstrated clear urine, diffuse small haemorrhagic patches within the bladder mucosa, normal ureteric orifices, and a normal urethra, with no other abnormality identified. The cystoscopy report did not specifically comment on trigonal involvement or the degree of mucosal oedema. No cystoscopic images were available, as images were not routinely captured during the procedure. As the cystoscopy was performed after clinical recovery, the findings may not fully reflect the severity of the initial inflammatory process observed during hospital admission.

Her case was subsequently discussed at the breast MDT meeting. Due to poor tolerance of the first chemotherapy cycle and significant anxiety regarding further toxicity, the team decided to proceed directly to surgery rather than continue neoadjuvant chemotherapy.

## Discussion

Haematuria and haemorrhagic cystitis are well-recognised adverse effects of oxazaphosphorine alkylating agents, such as cyclophosphamide and ifosfamide [1,2]. The underlying urothelial injury is primarily attributed to the accumulation of the toxic metabolite acrolein, which induces oxidative stress and inflammatory mediator release, ultimately leading to mucosal inflammation, oedema, and haemorrhage [2]. By contrast, the precise mechanism by which TCHP or its individual constituent agents cause urothelial injury remains poorly characterised [6-9].

To date, only a small number of cases of haematuria, cystitis, ureteritis, or related urothelial toxicities associated with TCHP have been reported in the literature. Table 1 summarises these reports together with selected contextual comparisons from related breast cancer systemic therapies and categorises them according to treatment exposure to facilitate distinction between TCHP-associated cases and non-TCHP-related urothelial adverse events [6-9]. The evidence specifically relating to TCHP-associated urothelial toxicity remains extremely limited. The exact mechanism underlying urothelial injury in our patient remains unclear. One possible explanation is the accumulation of an unidentified urotoxic metabolite arising from an individual component of the TCHP regimen, or from synergistic drug interactions between agents, resulting in inflammatory urothelial damage and haemorrhage. Based on previously reported cases with similar presentations, docetaxel has been postulated as the most likely causative agent within the TCHP regimen, although definitive causality remains difficult to establish in a multi-drug setting [6]. Histological confirmation was not obtained in our case, which limits definitive mechanistic interpretation.

**Table 1:** Published reports of TCHP-associated urothelial toxicity and selected contextual comparisons. Contextual comparison cases are included to illustrate potential mechanisms of urinary tract toxicity associated with individual components of breast cancer systemic therapy and should not be interpreted as evidence of TCHP-specific toxicity. Abbreviations: TCHP, docetaxel/carboplatin/trastuzumab/pertuzumab; HER2, human epidermal growth factor receptor 2; ER, oestrogen receptor; PR, progesterone receptor; CT, computed tomography; IHC, immunohistochemistry; NR, not reported.

Reference (year)	Age (years)	Sex	Category	Cancer diagnosis	Regimen and suspected agent	Clinical features and investigations	Management and outcome
Ramirez et al. (2025) [6]	43	Female	TCHP-associated	HER2+ ER− PR− T3N1 left invasive breast cancer	Neoadjuvant TCHP (docetaxel, carboplatin, trastuzumab, pertuzumab). Suspected agent: Docetaxel.	Gross haematuria with clot passage and suprapubic pain following the first TCHP cycle. CT urogram demonstrated minimal bladder wall thickening; no obstructive uropathy was identified.	Docetaxel substituted with paclitaxel. No further episodes of haematuria. The patient completed neoadjuvant chemotherapy.
Ramirez et al. (2025) [6]	53	Female	TCHP-associated	HER2+ ER+ PR+ T2N1 left invasive ductal carcinoma	Neoadjuvant TCHP (docetaxel, carboplatin, trastuzumab, and pertuzumab). Suspected agent: Docetaxel.	Gross haematuria with lower abdominal discomfort following the first TCHP cycle. Clinical and imaging findings consistent with acute inflammatory haemorrhagic cystitis.	Managed conservatively with nitrofurantoin and supportive care. Docetaxel substituted with paclitaxel, with subsequent resolution of haematuria.
Pastorello et al. (2022) [7]	NR	Female	TCHP-associated	HER2 3+ ER5% PR5% cT2N0M0 left invasive ductal carcinoma	Neoadjuvant TCHP (docetaxel, carboplatin, trastuzumab, and pertuzumab). Suspected agent: Carboplatin.	Gross haematuria and right flank pain. Imaging demonstrated mild-to-moderate dilatation of the right pyelocaliceal system with upper urinary tract obstruction secondary to clot formation. Findings were suggestive of haemorrhagic ureteritis.	Cystoscopy with clot evacuation and bladder irrigation. Carboplatin discontinued. Haematuria resolved. Completed neoadjuvant therapy with pathological complete response (ypT0N0).
Taj et al. (2011) [8]	34	Female	Carboplatin-associated (contextual comparison)	Basal-type breast carcinoma	Carboplatin-based chemotherapy (regimen not fully specified). Suspected agent: Carboplatin.	Gross haematuria developed two days after completion of carboplatin administration. No alternative urological pathology identified. Creatinine within normal limits; platelet count normal.	Managed conservatively with supportive treatment. Haematuria resolved with supportive management.
Cao et al. (2024) [9]	47	Female	HER2-directed therapy-associated (contextual comparison)	HER2+ pT1cN0M0 left invasive ductal carcinoma (Stage I)	Adjuvant paclitaxel + trastuzumab. Suspected agent: Trastuzumab (HER2 expression confirmed in urothelial cells on IHC).	Abdominal pain, lower back pain, dysuria, and gross haematuria. Anti-infective therapy ineffective. CT demonstrated extensive inflammatory exudation and oedema involving the bilateral ureters, renal pelvis, and bladder with dilatation and effusion (findings consistent with diffuse urothelial injury). Cystoscopy: congestion, oedema, and erosion of bladder epithelium. Biopsy: uroepithelial cell thinning and growth arrest. IHC: HER2 positivity in urothelial cells.	Treated with analgesia, dexamethasone, and cystoscopy. Treatment discontinued. Haematuria resolved during follow-up.

An important feature of this case was the rapid and marked clinical improvement following corticosteroid therapy, with resolution of haematuria, recovery of renal function, and stabilisation of haemoglobin occurring within 72 hours of treatment initiation. Although this observation is compatible with an inflammatory component to the underlying process, corticosteroid responsiveness alone does not establish an immune-mediated mechanism, and alternative explanations, including direct toxic urothelial injury, cannot be excluded. Furthermore, neither renal nor bladder biopsy was performed, and therefore alternative diagnoses, including drug-induced interstitial nephritis, IgA nephropathy, vasculitis, or other inflammatory processes, cannot be definitively excluded. The absence of histopathological confirmation represents an important limitation of this report and limits mechanistic interpretation. The report by Cao et al. described diffuse urothelial injury associated with trastuzumab-containing therapy and demonstrated HER2 expression within urothelial cells, suggesting one potential mechanism of treatment-related urothelial injury [9]. However, this report involved a non-TCHP regimen and is included only as a contextual comparison. Further studies are required to better define the pathophysiology of these rare adverse events.

Rare or poorly characterised toxicities associated with systemic anticancer therapy can lead to significant morbidity, treatment interruption, prolonged hospital admission, and delays in definitive oncological management. Increased clinical awareness of TCHP-associated urothelial toxicity is therefore essential to ensure timely recognition, appropriate investigation, and prompt management. Further research is needed to better characterise the mechanisms underlying treatment-related urothelial toxicity, which may help improve diagnostic pathways and management strategies. In selected cases, consideration should be given to the modification or substitution of the suspected causative agent where clinically appropriate.

## Conclusions

This case adds to the limited literature describing urothelial toxicity associated with TCHP and related breast cancer systemic therapies. Distinctive features included inflammatory cystitis, bilateral ureteritis with bilateral hydroureteronephrosis, acute kidney injury, and subsequent clinical improvement following corticosteroid therapy. Clinicians should be aware of these uncommon but potentially significant toxicities and consider them in the differential diagnosis of haematuria and acute kidney injury occurring after TCHP administration, particularly after exclusion of infection, obstruction, malignancy, and other common causes. Although corticosteroids may be considered in selected cases of presumed inflammatory urothelial toxicity, causality and the underlying pathological mechanism cannot be definitively established from a single case without histopathological confirmation. Further reports and studies are needed to better characterise the pathophysiology of TCHP-associated urothelial toxicity and to define optimal diagnostic and management strategies.
